# Predictors of Prediabetes Among Communities Without a Family History of Type 2 Diabetes Mellitus: A Case-Control Study

**DOI:** 10.7759/cureus.44131

**Published:** 2023-08-25

**Authors:** Iche A Liberty, Indri S Septadina, Muhammad Q Rizqie, Esti S Ananingsih, Hamzah Hasyim, Rico J Sitorus

**Affiliations:** 1 Biomedicine, Universitas Sriwijaya, Palembang, IDN; 2 Public Health and Community Medicine, Universitas Sriwijaya, Palembang, IDN; 3 Anatomy, Universitas Sriwijaya, Palembang, IDN; 4 Informatics Engineering, Universitas Sriwijaya, Palembang, IDN; 5 Epidemiology, Ministry of Health Palembang Health Polytecnic, Palembang, IDN; 6 Public Health, Universitas Sriwijaya, Palembang, IDN

**Keywords:** family history, predictors, risk, diabetes, prediabetes

## Abstract

Background

Prediabetes is the golden period to promote, prevent, or delay diabetes mellitus (DM) conversion. This study aims to assess the risk predictors associated with prediabetes among communities without a family history of type 2 DM (T2DM).

Methodology

This case-control study involved 570 participants (265 prediabetes cases and the same number of age-matched controls) in Palembang, Indonesia. Each participant is willing to take fasting blood glucose, lipid profile tests, and physical examinations.

Results

Multivariate analysis of this study revealed that significant risk predictors identified were occupation in the informal sector (_a_OR = 3.28; 95% CI = 1.64-6.58; p-value = 0.001), diastolic blood pressure of 80-89 mmHg (_a_OR = 2.18; 95% CI = 1.35-3.52; p-value = 0.001), diastolic blood pressure of 90-99 mmHg (_a_OR = 2.09; 95% CI= 1.15-3.82; p-value = 0.016), with an _a_OR = 5.80 (95% CI= 3.71-9.05; p-value <0.001). triglyceride-glucose index was the dominant risk predictor for prediabetes.

Conclusions

Knowing who is most vulnerable can guide the efficient allocation of promotion and prevention resources. This finding proves essential consideration for health promoters emphasizing a healthy diet and lifestyle by maintaining diastolic pressure and triglyceride glucose (TyG) index while considering the occupation in populations without a family history of T2DM.

## Introduction

Diabetes mellitus (DM) is a chronic disease characterized by a variety of metabolic disorders that impose a significant health burden on a country. In Indonesia, 7.6 million people suffer from DM which is characterized by hyperglycemia due to impaired insulin secretion and action [1]. Prediabetes or non-diabetic hyperglycemia is a term that refers to a high-risk metabolic state for DM, defined by a glycemic variable that is higher than normal but lower than the threshold for DM, namely the presence of impaired fasting glucose, and/or impaired glucose tolerance, and/or increased hemoglobin A1c (HbA1c) [2-3]. Individuals with prediabetes are at high risk of developing type 2 DM (T2DM) and its associated complications, with an estimated 5-10% of individuals with prediabetes developing T2DM each year [4-6]. Prediabetes carries some predictive power for macro-vascular disease (coronary disease, cerebrovascular disease, and peripheral vascular disease), but most of this association is mediated through metabolic syndrome [7]. These conditions occur together, increasing the risk of heart disease, stroke, and T2DM, and it is diagnosed when someone has three or more of these risk factors: high blood glucose, low levels of high-density lipoprotein (HDL) cholesterol in the blood, high levels of triglycerides in the blood, large waist circumference (WC), and high blood pressure.

The International Diabetes Federation estimated that 463 million adults aged 20-79 years are currently living with DM representing 9.3% of the world's population in this age group. The total number is predicted to rise to 578 million (10.2%) by 2030 and to 700 million (10.9%) by 2045. The number of deaths from DM and its complications in 2019 is estimated at 4.2 million [6]. Prediabetes will progress to T2DM in about 25% of subjects within three to five years, and 70% will progress in their lifetime [8]. Therefore, it is important to determine the predictors that can efficiently predict the development of prediabetes and identify the people who have a higher risk of prediabetes.

Being overweight, being physically inactive, getting older, having high blood pressure, and belonging to a racial or ethnic group with a family history of DM have long been identified as important risk factors for T2DM [9-10]. Although there are several genetic disorders associated with the risk of T2DM, the cause of most cases of the disease is multifactorial, involving the interaction of many genes and environmental or behavioral risk factors. Many studies have reported that there are still groups of people who have no family history but have DM or, conversely, who have a family history but do not have DM [11-12]. A study by Martha Rodríguez-Moran, et al. found that among 443 children and adolescents with a family history of DM, 390 (88.0%) had impaired fasting glucose; of them, 146 (37.4%), 79 (20.2%), and 165 (42.3%) were obese, overweight, and normal weight, respectively. Additionally, among 3280 children and adolescents without a family history, 62 (1.9%) had impaired fasting glucose; of these, 21 (33.9%), 14 (22.6%), and 27 (43.5%) were obese, overweight, and normal weight, respectively [13]. T2DM is largely inherited, but environmental factors such as diet and exercise can also affect how genes are expressed and whether DM develops. Genetic factors associated with DM can vary among individuals, and some people may have a combination of genetic variations that increase their risk, even without a family history. Currently, few studies have found a reliable way to predict which segments of the prediabetes population are most likely to develop DM. Understanding the risk factors associated with prediabetes is crucial for identifying individuals or groups who are most vulnerable to developing DM. However, currently, there are few reliable methods to predict which segments of the prediabetes population are most likely to progress to full-blown DM. Identifying high-risk populations and developing preventive strategies can help in managing and preventing the progression of DM, ultimately reducing the burden of the disease on individuals and communities. Knowing who the most vulnerable people are and where they live is the first step in managing and preventing DM. Therefore, a measure of vulnerability patterns that can be applied across communities and used to identify typical subgroups at risk is needed. This study aimed to identify the risk factors associated with prediabetes in communities without family history and to provide a scientific basis for prediabetes prevention and intervention.

## Materials and methods

Design and participants

We conducted a case-control study in Palembang, Indonesia, from July to November 2022. A total of 530 participants were enrolled in this study. Case pair matching is done prior to enrolment. The prediabetes determined by fasting plasma glucose is 100-125 mg/dl (5.6-6.9 mmol/l). Subjects with normal fasting plasma glucose levels were matched with prediabetes subjects living in the same village within three years. Community controls were selected from neighborhoods of prediabetes cases. The multistage cluster random sampling method was used to select a representative sample of the population. In the first stage, 2 of the 17 sub-districts were randomly selected from Palembang City, Seberang Ulu and Ilir. In the second stage, two of the five sub-districts in Seberang Ulu and two of the 12 in Seberang Ilir were randomly selected. In the third stage, 25% of the villages were randomly selected from each selected sub-district. In the final stage, all households with household members >18 years old were identified through fasting glucose test results.

Eligibility criteria are different for cases and controls for establishing glucose status. Inclusion of cases (fasting plasma glucose is 100-125 mg/dl (5.6-6.9 mmol/l)) and controls (fasting plasma glucose is between 70 mg/dl (3.9mmol/l) and 100mg/dl (5.6mmol/l)). The next eligibility criteria for cases and controls were as follows: (1) over 18 years old, (2) have no family history of T2DM (have no one or more first-degree relatives: parents, siblings, or children), and (3) agree to take fasting glucose, lipid profile tests, and physical examinations of their body weight, height, blood pressure, abdominal circumference, and WC. The exclusion criteria for cases and control were as follows: (1) currently taking oral hypoglycemic agents, (2) taking any drugs that would interfere with glucose and insulin metabolism or serum levels of HDL cholesterol, and (3) taking any obesity drugs.

Data collection, procedure, and measurements

All procedures carried out in this study involved human participants, in accordance with the ethical standards of the institutional research committee of Sriwijaya University with approval number 073-2022. All source documents, including questionnaires, were anonymized to ensure anonymity. We provided a standard questionnaire with an information sheet and consent form. Informed consent was taken from both cases and controls. Data were collected through interviews, physical examinations, and blood tests. The research team will go to the participant's home and complete a standard questionnaire with an information sheet and consent form. If the participants agreed, testing was performed the next day. After an overnight (minimum 8-12 hours) fasting, a blood sample was collected. Plasma glucose concentrations and serum total, low-density lipoprotein cholesterol (LDL-c), high-density lipoprotein cholesterol (HDL-c), and triglyceride concentrations were measured by standard routine laboratory procedures. The anthropometric evaluation was performed by a professional health trainer shortly after the removal of heavy clothes, belts, and shoes. Body weight was determined to the nearest 0.1 kg along with body height to the nearest 0.5 cm using the Tanita Scale (WB-800H plus digital model, Tanita Corporation of America, Inc., Illinois, USA). BMI was calculated by dividing body mass (kg) by height (m2). WC measurement was taken in the standing position at the midpoint between the iliac crest and the least palpable rib precisely using non-stretchable tape. Hip circumference (HC) was measured at the widest portion of the buttocks. The waist-to-hip ratio was calculated by dividing WC to HC. Optimal cut-offs estimated 90 cm in men and 80 cm in women to screen abdominal obesity [14].

The formula of the triglyceride glucose (TyG) index was as follows [15]:

Ln [TG (mg/dl) x FPG (mg/dl)]/2

The formula of lipid accumulation product (LAP) was as follows [16]:

For men, (WC [cm] − 65) × (triglyceride concentration [mM])

For women, WC [cm] − 58) × (triglyceride concentration [mM])

The formula of visceral adiposity index (VAI) was as follows [17]:

For men, [WC(cm)/39.68 + (1.88 × BMI)] × (TG(mmol/L)/1.03) × (1.31/HDL(mmol/L))

For women, [WC(cm)/36.58 + 1.89 × (BMI) ]× (TG(mmol/L)/0.81) × (1.52/HDL(mmol/L))

Data management and statistical analysis

The results were depicted as percentages and proportions for categorical variables. A comparison of predictors among prediabetes (cases) and normoglycemic (controls) was performed using logistic regression, and an OR with a 95% CI was calculated to study the association between predictors and outcomes. A p-value of <0.05 was considered significant. The data were analyzed using Stata version 15 (College Station, Texas, USA).

## Results

A total of 265 prediabetes and 265 normoglycemic were recruited at an overall case-to-control ratio of 1:1. The mean age of the normoglycemic was 39.60 ± 14.54 years (range = 18-67 years) and of the prediabetes was 42.19 ± 10.92 years (range = 18-67 years). Bivariate analysis in this study was obtained from the various predictors associated with prediabetes among communities without a family history of T2DM (Table 1). Men have a lower prevalence of prediabetes (43.40%) than women (56.60%). The analysis results (p = 0.538) showed that there was no significant association between sex and prediabetes. The formal sector of the cases (41,89%) is less than the controls (58,11%). Occupational factors (informal sector, formal sector, and unemployment) have a significant association with prediabetes.

**Table 1:** Predictors of prediabetes pertaining to risk factors LAP: lipid accumulation product, VAI: visceral adiposity index, TyG: triglyceride glucose, BMI: body mass index, mmHg: millimeters of mercury, WC: waist circumference

Variables	Prediabetes	Normoglycemic	Crude odds ratio (ORc) 95% CI; p-value
	n=265	n=265	
	n (%)	n (%)	
Sex			
Men	115 (43.40)	108 (40.75)	Reference category
Women	150 (56.60)	157 (59.25)	0.89 (0.62-1.28); 0.538
Occupation			
Student	19 (7.17)	58 (21.89)	Reference category
Informal sector	91 (34.34)	41 (15.47)	6.77 (3.59-12.79); <0.001
Formal sector	62 (23.40)	86 (32.45)	2.201 (1.19-4.06); 0.012
Unemployment	93 (35.09)	80 (30.19)	3.54 (1.95-6.45); <0.001
Systolic			
<120 mmHg	36 (13.58)	79 (29.81)	Reference category
120-139 mmHg	156 (58.87)	138 (52.08)	2.48 (1.57-3.91); <0.001
140-159 mmHg	63 (23.77)	40 (15.09)	3.45 (1.97-6.04); <0.001
>160 mmHg	10 (3.77)	8 (3.02)	2.74 (0.99-7.52); 0.050
Diastolic			
<80 mmHg	44 (16.60)	100 (37.70)	Reference category
80-89 mmHg	135 (50.90)	115 (43.40)	2.66 (1.73-4.11); <0.001
90-99 mmHg	61 (23.00)	40 (15.10)	3.46 (2.03-5.90); <0.001
>100 mmHg	25 (9.40)	10 (3.80)	5.68 (2.51-12.83); <0.001
BMI			
Underweight	8 (3.02)	12 (4.53)	Reference category
Normoweight	187 (70.57)	190 (71.70)	1.47 (0.59-3.69); 0.405
Overweight	26 (9.81)	30 (11.32)	1.3 (0.46-3.66); 0.620
Obese	44 (16.60)	33 (12.45)	2 (0.73-5.44); 0.175
Lipid index (LAP)			
<51.2	261 (98.49)	262 (98.87)	Reference category
>51.2	4 (1.51)	3 (1.13)	1.33 (0.29-6.03); 0.704
VAI			
<2.2	128 (48.30)	164 (61.89)	Reference category
>2.2	137 (51.70)	101 (38.11)	1.74 (1.23-2.46); 0.002
TyG index			
8-9	105 (39.62)	214 (80.75)	Reference category
9-10	160 (60.38)	51 (19.25)	6.39 (4.31-9.46); <0.001
WC			
Normal	156 (58.87)	157 (59.25)	Reference category
Central obesity	109 (41.13)	108 (40.75)	1.01 (0.71-1.43); 0.930
Waist-hip ratio			
Normal	22 (8.30)	24 (9.06)	Reference category
Obese	243 (91.70)	241 (90.94)	1.09 (0.60-2.01); 0.758

Another finding that is also important as a risk factor for prediabetes is the blood pressure status of the participants. Subjects with a systolic blood pressure of 120-139 mmHg (ORc = 2.48; 95% CI = 1.57-3.91; p = <0.001) and 140-159 mmHg (ORc = 3.45; 95% CI = 1.97-6.04; p= <0.001) were at risk of prediabetes compared to normoglycemic. Reciprocally, subjects with a diastolic blood pressure of 80-89 mmHg (ORc = 2.66; 95% CI = 1.73-4.11; p = <0.001), 140-159 mmHg (ORc = 3.46; 95% CI = 2.03-5.90; p = <0.001), and >100 mmHg (ORc = 5.68; 95% CI = 2.51-12.83, p = <0.001) were at risk of prediabetes compared to normoglycemic. While from the lipid index, the odds of prediabetes were higher for VAI >2.2 (ORc = 1.74; 95% CI = 1.23-2.46; p = 0.002), and TyG index 9-10 (ORc 6.39; 95% CI = 4.31-9.46, p = <0.001) compared to normoglycemic. BMI, physical activity, LAP, WC, and waist-hip ratio were not statistically significant predictors of risk of prediabetes (p >0.05). Multivariate logistic regression analysis was conducted to determine the dominant risk predictors for prediabetes as shown in Table 2. The adjusted odds of prediabetes were higher in the informal occupation sector (aOR = 3.28; 95% CI = 1.64-6.58; p-value = 0.001), diastolic 80-89 mmHg (aOR = 2.18; 95% CI = 1.35-3.52; p-value = 0.001), diastolic 90-99 mmHg (aOR = 2.09; 95% CI= 1.15-3.82; p-value = 0.016), with an aOR = 5.80 (95% CI = 3.71-9.05; p-value = <0.001). The TyG index was the dominant risk predictor for prediabetes.

**Table 2:** Multivariate logistic regression for the association of significant prediabetes risk predictors TyG: triglyceride glucose, mmHg: millimeters of mercury

Variables	Crude odds ratio (OR_c_) 95% CI; p-value	Adjusted odds ratio (OR_a_) 95% CI; p-value
Occupation		
Student	Reference category	Reference category
Informal sector	6.77 (3.59-12.79); <0.001	3.28 (1.64-6.58); 0.001
Formal sector	2.20 (1.19-4.06); 0.012	1.45 (0.75-2.79); 0.265
Unemployment	3.54 (1.95-6.45); <0.001	1.22 (0.61-2.41); 0.569
Diastolic		
<80 mmHg	Reference category	Reference category
80-89 mmHg	2.66 (1.73-4.11); <0.001	2.18 (1.35-3.52); 0.001
90-99 mmHg	3.46 (2.03-5.90); <0.001	2.09 (1.15-3.82); 0.016
>100 mmHg	5.68 (2.51-12.83); <0.001	2.06 (0.77-4.69); 0.169
TyG index		
8-9	Reference category	Reference category
9-10	6.39 (4.31-9.46); <0.001	5.80 (3.71-9.05); <0.001

## Discussion

Prediabetes has been identified as a precursor to T2DM, and with the increasing prevalence of T2DM, timely identification of prediabetes risk factors is essential for primary prevention and it serves as a tool. Several previous studies have shown that a variety of factors at the individual, household, and community levels can influence prediabetes and DM. People with lower education reported having a prevalence of not only DM but also hypertension [18-19]. Our study found that people who do less physical work have a higher likelihood of developing hypertension and DM. Thus, an initiative program for monitoring the progression of hypertension and/or DM for those with education and working in professional occupations that do not require physical work, such as in the formal sector, must differ from the informal sector.

An important finding as a risk factor for prediabetes was the blood pressure status of the participants. Impaired glucose metabolism and insulin resistance are the main pathophysiology of T2DM. Several studies have analyzed the association between insulin resistance and changes in heart function with diastolic dysfunction. The findings of this study showed that diastolic had a significant relationship with prediabetes. A study conducted by Fontes et al. (2015) found that individuals with higher insulin resistance had worse diastolic function parameters and a significantly increased risk of left ventricular (LV) diastolic dysfunction, independent of other determinants of diastolic function [20]. According to Di Pino et al. (2017), subjects with HbA1c categorized as prediabetes exhibited higher left atrial volume (LAV) [21]. Dinh et al. demonstrated an increase in LAV from normal glucose tolerance to impaired glucose tolerance and T2DM and that HbA1c was significantly correlated with LAV and E/e0 ratios, parameters indicating LV diastolic dysfunction with increased filling pressure, even in subjects without a history of DM. The authors suggested that the LAV reflects the cumulative effect of different long-duration contributors to LV diastolic function and is less susceptible to acute changes in pre-load and after-load, which may have acute effects on diastolic function [22].

The pathophysiologic defects underlying prediabetes include insulin resistance, alpha- and beta-cell dysfunction, increased lipolysis, inflammation, and oxidative stress [23-24]. A study showed that in patients with coronary artery disease combined with both prediabetes and hypertension, their blood glucose and blood pressure levels are significantly higher than those with either prediabetes or hypertension alone, suggesting that prediabetes and hypertension have a synergistic effect on cardiovascular outcomes [25]. A hyperglycemic environment induces hypertension, which increases the disturbed renal perfusion pressure and indirectly causes microvascular damage in renal arteries, and glomerular and tubulointerstitial capillaries. Upregulated sodium-glucose co-transporter expression promotes glucose uptake, which affects the tubular-glomerular feedback mechanism, leading to glomerular hypertension [26]. Plasma levels of kallikrein, thrombin, and coagulation factor VII are elevated in prediabetes, which can lead to hypertension and cardiovascular disease [27].

The results of this study also indicate that the TyG index is one of the significant risk markers for prediabetes in multivariate analysis. Several cohort studies found an association between the TyG index and the increased risk of prediabetes. The normal cut-off values reported for the TyG in the literature are roughly around 4 and 8 [28-29]. Insulin resistance is the main pathophysiological basis for dysglycemia, and this may explain the reason the TyG index has a higher predictive ability for prediabetes. TyG is a composite index of fasting triglycerides and glucose. Glycerol and fatty acids are two products of triglyceride lipolysis that can enhance hepatic gluconeogenesis [30]. The results of both animal and human studies also showed that a high intake of saturated fat is associated with insulin resistance and the development of T2DM because an uncontrolled state of insulin resistance leads to a higher risk of developing the disease. The TyG index has several advantages over other insulin resistance screening tools in terms of cost and accessibility. Fasting triglycerides and blood glucose levels are the two measurements required to generate the TyG index. This makes it an easy-to-use tool for screening in the community, as it requires no specialized equipment or training. The TyG index is a low-cost parameter that can be easily incorporated into routine blood tests, especially in resource-constrained environments. By using the TyG index in community screening, healthcare providers can identify individuals with insulin resistance and tailor interventions based on their risk profile. These interventions may include lifestyle modifications, dietary changes, and pharmacological interventions targeted at improving insulin sensitivity and preventing the development of associated health conditions. However, we cannot rule out the possibility that these observations could be confounded by other dietary components and various lifestyle habits. While focusing only on fasting glucose may yield valuable findings, it is possible that some individuals at risk of prediabetes may not be well detected through this criterion alone. The use of more than one diagnostic criterion is highly recommended for more accuracy. This study can be used as a reference for planning the promotion and prevention of prediabetes programs that differ from other common programs. To reduce the prevalence of T2DM, we need to know the prediabetes risk factors. Many studies found policies or programs to prevent DM or other non-communicable diseases. However, the studies did not pay attention to specific segments, such as those who do not have a prior family history. This is important because the population with a family history of T2DM will be more attentive to the risk factors in contrast to the population segment without a family history with lots of risky lifestyle changes.

## Conclusions

This finding highlights the importance of health promoters emphasizing a healthy diet and lifestyle by maintaining diastolic pressure and TyG index while taking into account occupation in populations without a family history of T2DM. Designing programs that address the unique challenges and prevalent risk factors can help increase awareness and promote healthy behaviors. By considering these specific population segments and their distinct risk profiles, promotion and prevention programs can be designed to be more targeted, culturally sensitive, and effective. This comprehensive approach can contribute to reducing the prevalence of prediabetes and T2DM, as well as other cardiometabolic diseases.
